# Detailed immunohistochemical characterization of temporal and spatial progression of Alzheimer's disease-related pathologies in male triple-transgenic mice

**DOI:** 10.1186/1471-2202-9-81

**Published:** 2008-08-12

**Authors:** Michael A Mastrangelo, William J Bowers

**Affiliations:** 1 Department of Neurology, University of Rochester Medical Center, Rochester, NY 14642, USA; 2 Department of Microbiology and Immunology, University of Rochester Medical Center, Rochester, NY 14642, USA; 3 Center for Neural Development and Disease, University of Rochester Medical Center, Rochester, NY 14642, USA

## Abstract

**Background:**

Several transgenic animal models genetically predisposed to develop Alzheimer's disease (AD)-like pathology have been engineered to facilitate the study of disease pathophysiology and the vetting of potential disease-modifying therapeutics. The triple transgenic mouse model of AD (3xTg-AD) harbors three AD-related genetic loci: human PS1^M146V^, human APP^swe^, and human tau^P301L^. These mice develop both amyloid plaques and neurofibrillary tangle-like pathology in a progressive and age-dependent manner, while these pathological hallmarks are predominantly restricted to the hippocampus, amygdala, and the cerebral cortex the main foci of AD neuropathology in humans. This model represents, at present, one of the most advanced preclinical tools available and is being employed ever increasingly in the study of mechanisms underlying AD, yet a detailed regional and temporal assessment of the subtleties of disease-related pathologies has not been reported.

**Methods and results:**

In this study, we immunohistochemically documented the evolution of AD-related transgene expression, amyloid deposition, tau phosphorylation, astrogliosis, and microglial activation throughout the hippocampus, entorhinal cortex, primary motor cortex, and amygdala over a 26-month period in male 3xTg-AD mice. Intracellular amyloid-beta accumulation is detectable the earliest of AD-related pathologies, followed temporally by phospho-tau, extracellular amyloid-beta, and finally paired helical filament pathology. Pathology appears to be most severe in medial and caudal hippocampus. While astrocytic staining remains relatively constant at all ages and regions assessed, microglial activation appears to progressively increase temporally, especially within the hippocampal formation.

**Conclusion:**

These data fulfill an unmet need in the ever-widening community of investigators studying 3xTg-AD mice and provide a foundation upon which to design future experiments that seek to examine stage-specific disease mechanisms and/or novel therapeutic interventions for AD.

## Background

Alzheimer's disease (AD) represents the most common age-related neurodegenerative disorder and cause of dementia worldwide. The prevalence of AD is predicted to increase significantly to affect over 100 million people worldwide by the year 2050 [1]. With this dire prediction, it has become imperative to dissect the pathophysiologic mechanisms intrinsic to AD in an effort to eventually devise disease course-modifying therapies. Individuals afflicted with AD harbor two pathological signatures within their brains: extracellular amyloid plaques and neurofibrillary tangles (NFTs), which are identifiable only upon post-mortem examination. Extracellular plaques are comprised of proteinaceous aggregates of amyloid beta (Aβ) peptides, ubiquitin, various proteoglycans, proteases, serum-related molecules, as well as numerous other proteins [2]. The major amyloidogenic components of plaque, Aβ 1–40 and 1–42 peptides, are the proteolytically liberated products that arise from the enzymatic processing of amyloid precursor protein (APP), a type 1 transmembrane protein. NFTs are the result of intraneuronal hyperphosphorylated paired helical filaments of the microtubule-associated protein tau. The seminal work by Drs. Heiko and Eva Braak demonstrated that these pathologies proceed in a definable temporal and spatial pattern within the human brain [3]. Stage A of amyloid accumulation represents the presence of amyloid patches in the basal neocortex and in poorly myelinated temporal areas such as perirhinal and entorhinal areas; the spreading of amyloid deposition to neocortical areas and the hippocampus is indicative of Stage B, while Stage C includes appearance of amyloid deposits in highly myelinated areas of the cortex and neocortex. The evolution of NFTs in the AD brain proceeds through six distinct stages that to some extent overlap with those of amyloid deposition. Stage I is defined by NFT appearance in cell projections comprising the trans-entorhinal region of the temporal lobe, whereas evidence of NFT pathology in the entorhinal region, hippocampus/temporal pro-neocortex is indicative of Stages II and III, respectively. Stages IV-VI of NFT formation includes progression to the neocortex and areas adjoining the neocortex.

To elucidate the varying pathophysiologic mechanisms underlying AD progression and to assess potential disease-modifying therapeutics in a preclinical *in vivo *setting, investigators have turned to transgenic mouse models harboring mutated human genes associated with the familial forms of AD. Although no single transgenic model recapitulates the human disease in all aspects of neuropathology and behavior, some assumptions can be made as to which model best fits specific criteria of AD. Amyloid-based transgenic mouse models exist that overexpress wild-type or mutant forms of APP (i.e., Tg2576; [4]), leading to extracellur Aβ peptide accumulation into plaque-like deposits, synaptic loss, microgliosis, astrocytosis, and cerebrovascular angiopathy [5,4,10]. Most of these models exhibit differential behavioral phenotypes related to significant learning and memory impairment, spatial deficits, and at times, increased aggression. At least nine transgenic mouse models have been created to study consequences of pathogenic tau expression [11-16]. All models show pathology of varying severity, including models overexpressing normal human tau.

The triple-transgenic Alzheimer's disease (3xTg-AD) mouse, created in the laboratory of Dr. Frank LaFerla, represents one of the most state-of-the-art and biologically relevant mouse model for AD described to date. The 3xTg-AD mouse model was generated by co-microinjection of the human APP^swe ^and tau^P301L ^genes, both under the transcriptional control of a modified Thy1.2 promoter, into single-cell homozygous mutant PS1^M146V ^knock-in mouse embryos [17]. These mice develop intracellular Aβ, amyloid plaques and NFTs in a progressive and age-related pattern, where the pathologies are predominantly restricted to the hippocampus, amygdala, and the cerebral cortex [18]. These mice also exhibit deficits in synaptic functioning, including long-term potentiation (LTP) [17], and learning/memory behaviors that are similarly manifested in an age-dependent manner [19]. These early papers describing the derivation and initial characterization of pathological progression in 3xTg-AD mice was limited in terms of temporal and regional evolution of particular AD-related hallmarks. Moreover, it has been difficult to glean from those reports as to whether one gender was exclusively studied or whether experimental groups consisting of mixed genders were employed [17].

A detailed regional and temporal assessment of the subtleties of disease-related brain pathologies that arise in 3xTg-AD mice over much of their lifespan has yet to be reported. Absence of such information may lead one to improperly initiate long-term experiments designed to address specific hallmarks of AD-related pathology. To that end, we have systematically examined the temporal and spatial progression of human APP^swe ^transgene expression, appearance of intracellular and extracellular Aβ_1–42_, human tau^P301L ^transgene expression, appearance of pathogenic phospho-tau, and evidence of microglial activation and astrogliosis in male 3xTg-AD mice from 2 to 26 months of age.

## Results

### Human APP^swe ^Transgene Expression

**Figure 1:**
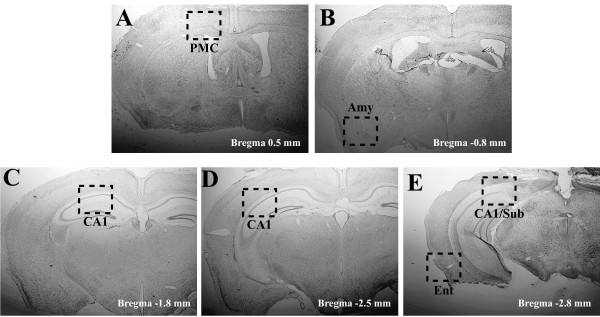
**Representative Nissl-stained brain sections from 3xTg-AD mice depicting regions examined by immunohistochemistry in this study**. Coronal mouse brain sections (30 μm) were prepared from 3xTg-AD mice sacrificed at 6 months of age and were processed for Nissl staining. Primary motor cortex (PMC) at Bregma 0.5 mm (**A**), amygdala (Amy) at Bregma -0.8 mm (**B**), CA1 hippocampal sections at Bregma -1.8 mm (**C**), at Bregma -2.5 mm (**D**), and at Bregma -2.8 mm (**E**), as well as entorhinal cortex (Ent) at Bregma -2.8 mm (**E**) are outlined by dotted boxes to illustrate the sub-regions of the brains examined in this study. Photomicrographs were obtained at 1.25×.

**Figure 2:**
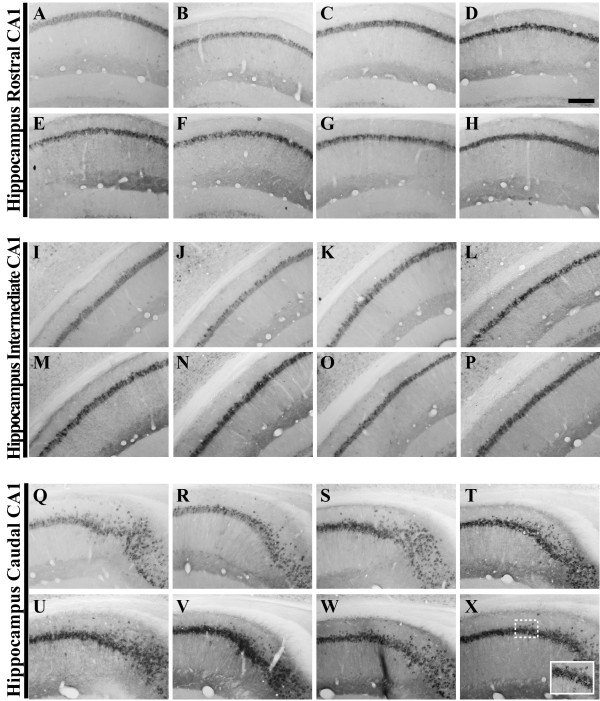
**Human amyloid precursor protein transgene expression is readily detectable within the pyramidal cell layer of 3xTg-AD mouse hippocampus from 2 to 26 months of age**. Coronal mouse brain sections (30 μm) were prepared from 3xTg-AD mice sacrificed at 2 (**A, I, Q**), 3 (**B, J, R**), 6 (**C, K, S**), 9 (**D, L, T**), 12 (**E, M, U**), 15 (**F, N, V**), 18 (**G, O, W**), and 26 months of age (**H, P, X**) and were processed for immunohistochemistry to detect human amyloid precursor protein (hAPP) A4 using the Y188 monoclonal antibody. CA1 hippocampal sections at Bregma -1.8 mm (**A–H**), at Bregma -2.5 mm (**I-P**), and at Bregma -2.8 mm (**Q-X**), were examined for regional and temporal patterns of hAPP^swe ^transgene expression and photomicrographs were obtained at 10×. The inset in panel **X **represents a 40× digitally magnified image of the photomicrograph for better visualization of stained cell morphology. Scale bar in **D **represents 200 μm.

**Figure 3:**
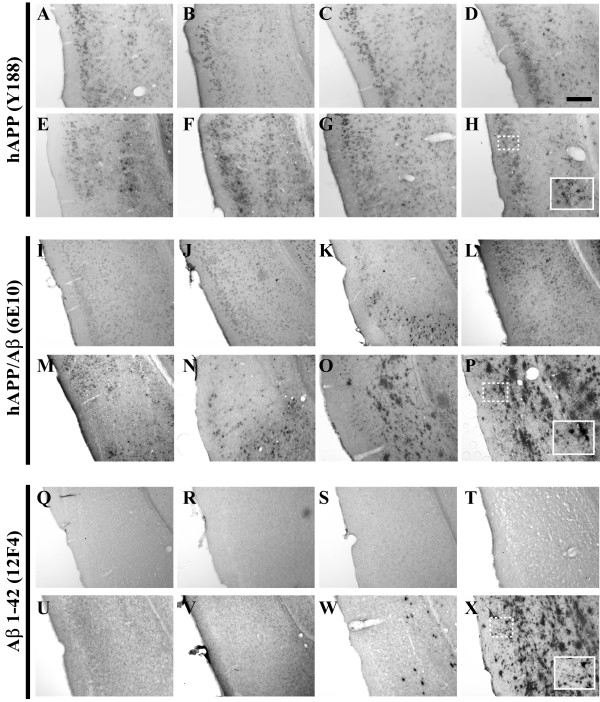
**Entorhinal cortex-resident human APPswe transgene expression and Aβ peptide deposition evolve on similar timescales as observed in the 3xTg-AD hippocampus**. Coronal mouse brain sections (30 μm) were prepared from 3xTg-AD mice sacrificed at 2 (**A, I, Q**), 3 (**B, J, R**), 6 (**C, K, S**), 9 (**D, L, T**), 12 (**E, M, U**), 15 (**F, N, V**), 18 (**G, O, W**), and 26 months of age (**H, P, X**) and were processed for immunohistochemistry to detect the human Swedish mutant amyloid precursor protein (hAPP^swe^) transgene product using the Y188 monoclonal antibody (**A–H**), human amyloid precursor protein (hAPP) and Aβ peptides using the 6E10 monoclonal antibody (**I-P**), and extracellular Aβ_1–42 _deposition using the 12F4 monoclonal antibody (**Q-X**). Entorhinal cortex was examined for patterns of immunopositivity and photomicrographs were obtained at 10×. The insets in panels **H**, **P**, and **X **represent 40× digitally magnified images of designated photomicrographs for better visualization of immunopositive cell/pathology. Scale bar in **D **represents 200 μm.

**Figure 4:**
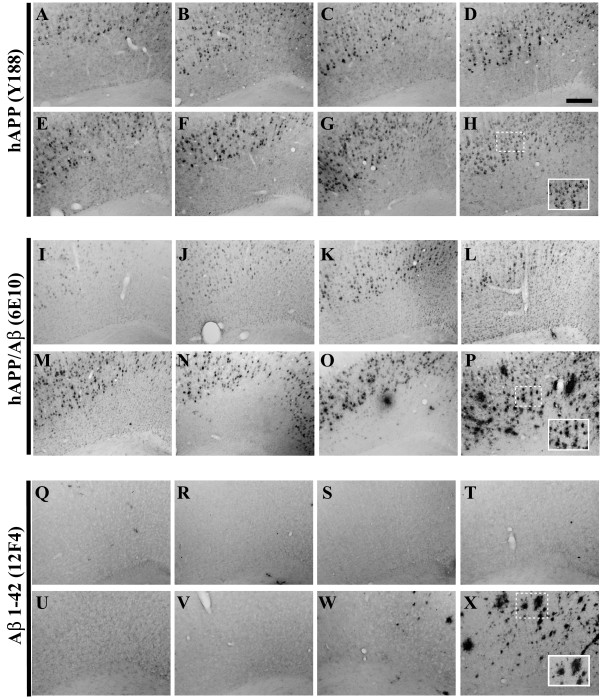
**Primary motor cortex-resident human APPswe transgene expression and Aβ peptide deposition evolve on similar timescales as compared to the 3xTg-AD hippocampus**. Coronal mouse brain sections (30 μm) were prepared from 3xTg-AD mice sacrificed at 2 (**A, I, Q**), 3 (**B, J, R**), 6 (**C, K, S**), 9 (**D, L, T**), 12 (**E, M, U**), 15 (**F, N, V**), 18 (**G, O, W**), and 26 months of age (**H, P, X**) and were processed for immunohistochemistry to detect the human Swedish mutant amyloid precursor protein (hAPP^swe^) transgene product using the Y188 monoclonal antibody (**A–H**), human amyloid precursor protein (hAPP) and Aβ peptides using the 6E10 monoclonal antibody (**I-P**), and extracellular Aβ_1–42 _deposition using the 12F4 monoclonal antibody (**Q-X**). Primary motor cortex was examined for patterns of immunopositivity and photomicrographs were obtained at 10×. The insets in panels **H**, **P**, and **X **represent 40× digitally magnified images of designated photomicrographs for better visualization of immunopositive cell/pathology. Scale bar in **D **represents 200 μm.

**Figure 5:**
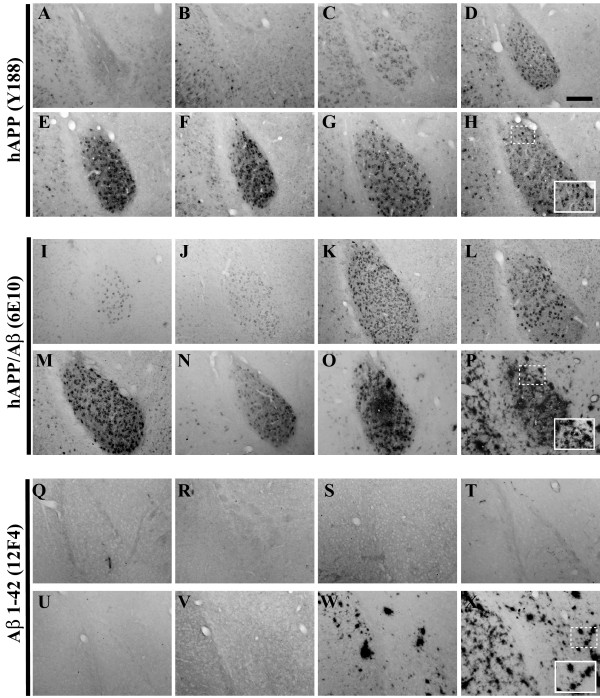
**Amygdala-resident human APPswe transgene expression is slightly delayed, while Aβ peptide deposition evolves on a similar timescale as compared to the 3xTg-AD hippocampus**. Coronal mouse brain sections (30 μm) were prepared from 3xTg-AD mice sacrificed at 2 (**A, I, Q**), 3 (**B, J, R**), 6 (**C, K, S**), 9 (**D, L, T**), 12 (**E, M, U**), 15 (**F, N, V**), 18 (**G, O, W**), and 26 months of age (**H, P, X**) and were processed for immunohistochemistry to detect the human Swedish mutant amyloid precursor protein (hAPP^swe^) transgene product using the Y188 monoclonal antibody (**A–H**), human amyloid precursor protein (hAPP) and Aβ peptides using the 6E10 monoclonal antibody (**I-P**), and extracellular Aβ_1–42 _deposition using the 12F4 monoclonal antibody (**Q-X**). Amygdala was examined for patterns of immunopositivity and photomicrographs were obtained at 10×. The insets in panels **H**, **P**, and **X **represent 40× digitally magnified images of designated photomicrographs for better visualization of immunopositive cell/pathology. Scale bar in **D **represents 200 μm.

In the present study, we sought to immunohistochemically document the temporal and regional evolution of AD-related transgene expression, amyloid deposition, tau phosphorylation, astrogliosis, and microglial activation throughout the hippocampus, amygdala, primary motor cortex, and entorhinal cortex over a 26-month period in male 3xTg-AD mice (Antibodies employed in this study are shown in Table 1 and Nissl-stained brain regions of interest depicted in Figure 1). To sufficiently garner detailed insight into how AD-related pathologies arise in 3xTg-AD mice, animals were sacrificed at 2, 3, 6, 9, 12, 15, 18, and 26 months of age for subsequent immunohistochemical processing (N = 4 per time point). Amyloid pathology that develops in these mice derives from the proteolytic processing of the human APP transgene product that harbors the Swedish double mutation (K595N/M596L; [20]) and M146V knock-in mutation in presenilin 1 [21,22] that, in combination, lead to the marked overproduction and progressive accumulation of the fibrillogenic peptide, Aβ_1–42 _[23]. The hAPP^swe ^transgene, as well as the tau^P301L ^transgene, is under the transcriptional control of Thy1.2 gene promoter, which results in transgene expression specifically within neuronal populations beginning early in post-natal development and continuing into adulthood [24]. Human APP^swe ^transgene expression, as assessed using the APP Y188 antibody (AbCam), which recognizes the NPXY amino acid motif of the hAPP protein localized amino terminal to the cleavage fragment of Aβ, was detectable in 3xTg-AD mouse brain beginning at the 2-month time point throughout the pyramidal neurons of the hippocampus (rostral, intermediate, and caudal; Figure 2), layer II and III neurons of the entorhinal cortex (Figure 3A–H), and primary motor cortex (Figure 4A–H). Staining intensities for hAPP^swe ^transgene product qualitatively appear to stabilize from 6 months and older in all of the regions of the hippocampus and entorhinal cortex examined (Figures 2 and 3A–H). Interestingly, in the amygdala, hAPP^swe ^expression is not detected by the Y188 antibody consistently until 6 months of age (Figure 5A–H).

**Table 1:** Antibodies Employed in Present Study

**Target Epitope**	**Antibody (*Supplier*)**	**Reference**
hAPP	Rabbit monoclonal clone Y188	-
Amyloid precursor protein A4, corresponding to the NPXY motif of hAPP	*(Abcam)*	
hAPP/Aβ	Mouse monoclonal clone 6E10	Götz J, et al. [70]
Aβ amino acid residues 1–16; also cross-reacts with hAPP	*(Signet)*	Hock C, et al. [71]
		Oddo S, et al. [72]
hAβ 1–42 (extracellular) C-terminus of Aβ 1–42	Mouse monoclonal clone 12F4	Parvathy, S., et al. [73]
	*(Covance/Signet)*	
hAβ 1–42 (intracellular) C-terminus of Aβ 1–42	Rabbit polyclonal anti Aβ 1–42	D'Andrea, et al [27]
	*(Biosource/Invitrogen)*	
hTau	Mouse monoclonal HT7	Oddo et al [18]
Human Tau amino acid residues 159–163	*(Pierce)*	Mercken M et al [74]
Phosphorylated Tau	Mouse monoclonal AT180	Greenberg and Davies [39]
Human Tau phosphorylated residue Thr231	*(Pierce)*	Oddo S et al. [18]
Paired Helical Filaments	Monoclonal mouse anti-PHF-1	Ksiezak-Reding, et al. [37]
Human Tau phosphorylated on amino acid residues Ser396 and Ser404 associated with paired helical filaments	*(Dr. Peter Davies, Albert Einstein School of Medicine)*	Clinton, et al. [68]
F4/80	Rat anti mouse F4/80	Janelsins et al. [52]
	*(AbD Serotec)*	
Cell surface glycoprotein on mature macrophages that is a member of the EGF-TM7 family		
GFAP	Rabbit polyclonal anti-GFAP	Shaftel S, et al. [75]
Cell surface marker (glial fibrillary acidic protein) for mature astrocytes	*(Dako Cytomation)*	

### Patterns of Intracellular and Extracellular Aβ Peptide Accumulation

**Figure 6:**
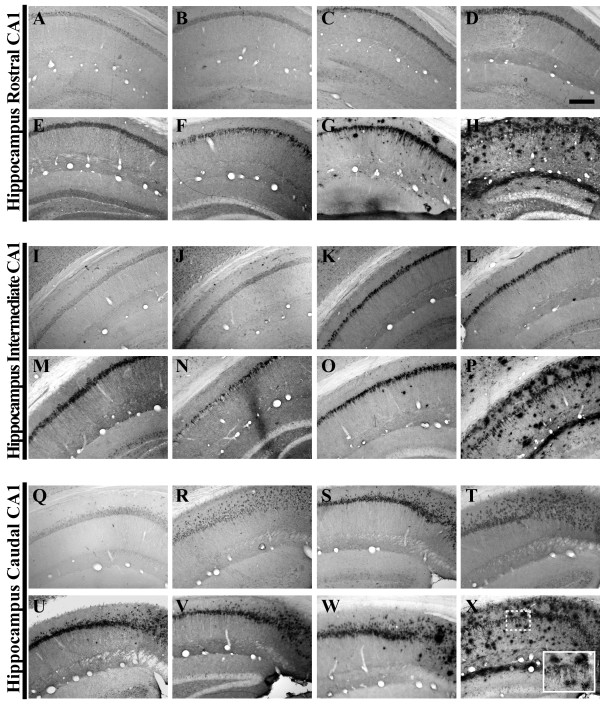
**6E10 immunohistochemistry reveals differential cell-associated hAPP/Aβ accumulation patterns and markedly late-stage extracellular plaque deposition in 3xTg-AD mouse hippocampus**. Coronal mouse brain sections (30 μm) were prepared from 3xTg-AD mice sacrificed at 2 (**A, I, Q**), 3 (**B, J, R**), 6 (**C, K, S**), 9 (**D, L, T**), 12 (**E, M, U**), 15 (**F, N, V**), 18 (**G, O, W**), and 26 months of age (**H, P, X**) and were processed for immunohistochemistry using the 6E10 monoclonal antibody to detect both human amyloid precursor protein (hAPP) and Aβ peptides. CA1 hippocampal sections at Bregma -1.8 mm (**A–H**), at Bregma -2.5 mm (**I–P**), and at Bregma -2.8 mm (**Q-X**), were examined for regional and temporal patterns of hAPP^swe ^transgene/Aβ peptide expression and photomicrographs were obtained at 10×. The inset in panel **X **represents a 40× digitally magnified image of the photomicrograph for better visualization of stained cell morphology. Scale bar in **D **represents 200 μm.

Assessment of Aβ peptide deposition was performed using two antibodies with differing specificity: 6E10, which recognizes amino acid residue 1–16 of beta-amyloid, but also reacts with that identical epitope within non-proteolytically processed hAPP; and monoclonal antibody 12F4, which is specifically reactive to the C-terminus of Aβ_1–42_. The expression of APP/Aβ as detected by 6E10 reveals similar cell-associated patterns of staining to those observed in sections stained with the hAPP-specific antibody (Y188) through 12 months of age (Figures 3I–P, 4I–P, 5I–P, and 6). The co-staining of APP/Aβ begins to appear in CA1 neurons at approximately 3 months of age in the caudal most region of the hippocampus (Figure 6R), neurons in layer II and III of the entorhinal cortex (Figure 3J), and neurons of the primary motor cortex (Figure 4J). Evidence of 6E10 staining in the amygdala does not become appreciable until 6 months of age (Figure 5K). The intracellular expression of APP/Aβ is confined to the pyramidal layer of the hippocampus and all other brain regions until development of extracellular deposition begins to be apparent at ages greater than 15 months (Figures 3O, 4O, 5O, and 6V, G, O). With all cell-associated immunopositive signal, APP/Aβ staining is limited to the cell bodies, with little to no staining of fibers residing in the radiatum of the hippocampus. Plaques that appear at 18 and 26 months of age are found in the stratum oriens, stratum lucidium and radiatum of the hippocampus, and layers V and VI of the entorhinal cortex, with a majority of the extracellular plaques developing within the subiculum.

**Figure 7:**
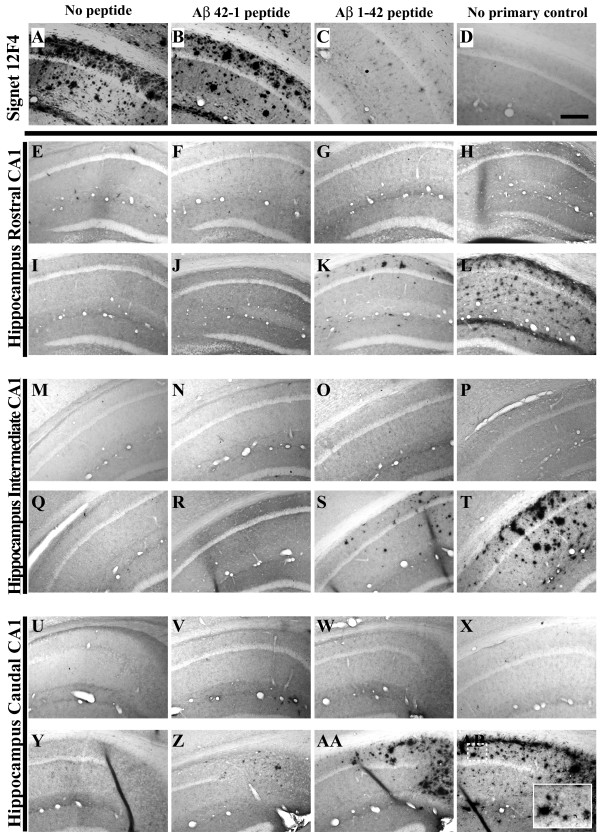
**Extracellular Aβ _1–42 _deposition is not immunohistochemically detectable in male 3xTg-AD mouse hippocampus until 15 months of age**. A monoclonal antibody specific for human Aβ_1–42 _(12F4; Signet) was incubated with 20 month-old 3xTg-AD mouse brain sections alone (**A**), or with a 200-fold molar excess of the cognate reverse peptide (**B**), forward peptide (**C**), or no-primary control (**D**) according to a protocol designed to detect extracellular Aβ_1–42_. Coronal mouse brain sections (30 μm) were prepared from 3xTg-AD mice sacrificed at 2 (**E, M, U**), 3 (**F, N, V**), 6 (**G, O, W**), 9 (**H, P, X**), 12 (**I, Q, Y**), 15 (**J, R, Z**), 18 (**K, S, AA**), and 26 months of age (**L, T, AB**) and were processed for immunohistochemistry using the 12F4 antibody to detect extracellular Aβ_1–42 _peptide accumulation. CA1 hippocampal sections at Bregma -1.8 mm (**E-L**), at Bregma -2.5 mm (**M-T**), and at Bregma -2.8 mm (**U-AB**), were examined for regional and temporal patterns of extracellular Aβ_1–42 _deposition and photomicrographs were obtained at 10×. The inset in panel **AB **represents a 40× digitally magnified image of the photomicrograph for better visualization of stained cell morphology. Scale bar in **D **represents 200 μm.

To confirm that the extracellular plaque-like deposition pattern was the result of accumulated Aβ_1–42 _peptide, adjacent sections were stained with the anti-Aβ_1–42 _antibody 12F4 (Figures 3Q–X, 4Q–X, 5Q–X, and 7). Using a peptide competition experiment, this antibody was shown to specifically recognize extracellular Aβ_1–42 _(Figure 7A-D). It is readily apparent that Aβ_1–42_-reactive deposits accumulate in the hippocampus starting at the subiculum/CA1 interchange at 15 months of age (Figure 7Z). Prior to this age, Aβ_1–42 _accumulation in the hippocampus is below the threshold of detection for this antibody in male 3xTg-AD mice. Interestingly the more rostral areas of the hippocampus even at the 15-month time point do not show any Aβ_1–42 _reactivity (Figure 7J, R). By 18 months of age, the Aβ_1–42 _burden is rather significant with large dense plaques apparent in the caudal hippocampus at the area of the subiculum/CA1 interchange (Figure 7AA) with smaller deposits appearing in the stratum oriens and radiatum flanking the pyramidal layer of the hippocampus (Figure 7K, S). By 26 months the dense cored extracellular Aβ_1–42 _deposits have spread throughout the hippocampus. Intriguingly, Aβ_1–42 _reactivity is lacking in the cells that comprise the pyramidal layer of the CA1, which robustly stained with 6E10 at the same age (Figure 6H, P, X). Patterns of Aβ_1–42 _deposition in the entorhinal cortex begins in the deeper layers at 18 months of age (Figure 3W) and spreads to the more superficial layers, encompassing the entire entorhinal cortex by 26 months (Figure 3X). Similar observations of extracellular Aβ 1–42 deposition can be seen at 18 and 26 months in amygdala and primary motor cortex (Figures 4W, X, and 5W, X).

**Figure 8:**
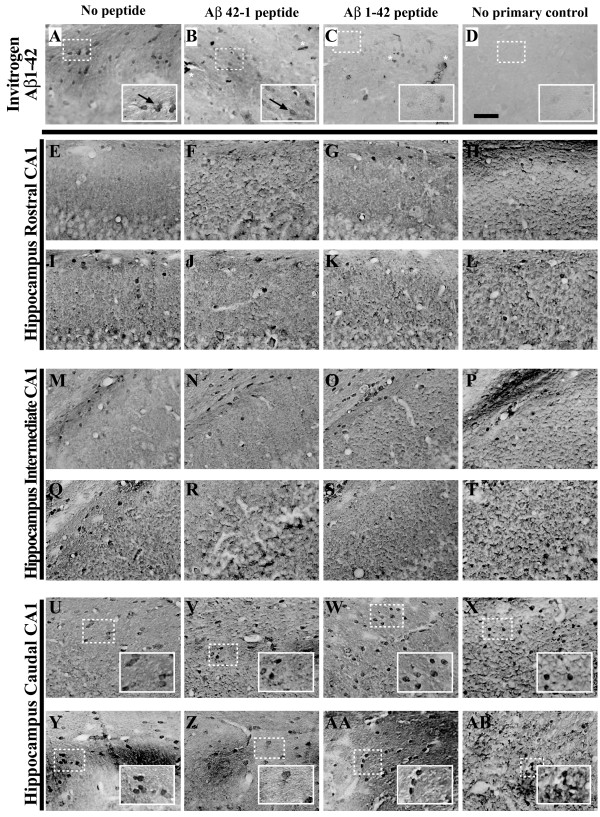
**Intracellular Aβ _1–42 _accumulation is immunohistochemically detectable by 3 months of age in 3xTg-AD mouse hippocampus**. A polyclonal antibody specific for human Aβ_1–42 _(Biosource/Invitrogen) was incubated with 20 month-old 3xTg-AD mouse brain sections alone (**A**), or with a 200-fold molar excess of the cognate reverse peptide (**B**), forward peptide (**C**), or no-primary control (**D**) according to a protocol designed to detect intracellular Aβ_1–42 _[27]. Arrows indicate immunopositive cells, while "*" depicts non-specific signal due to precipitant. Coronal mouse brain sections (30 μm) were prepared from 3xTg-AD mice sacrificed at 2 (**E, M, U**), 3 (**F, N, V**), 6 (**G, O, W**), 9 (**H, P, X**), 12 (**I, Q, Y**), 15 (**J, R, Z**), 18 (**K, S, AA**), and 26 months of age (**L, T, AB**) and were processed for immunohistochemistry to detect intracellular Aβ_1–42 _peptide accumulation using the Biosource/Invitrogen anti-Aβ_1–42 _polyclonal antibody. CA1 hippocampal sections at Bregma -1.8 mm (**E-L**), at Bregma -2.5 mm (**M-T**), and at Bregma -2.8 mm (**U-AB**), were examined for regional and temporal patterns of intracellular Aβ_1–42 _and photomicrographs were obtained. The insets in panels **U-AB **represent digitally magnified images of designated photomicrographs for more optimal visualization of stained cell morphology. Scale bar in **D **represents 50 μm.

An early role of intracellular Aβ in neuronal dysfunction has been proposed (reviewed by [25]). Oddo et al. previously reported 3xTg-AD mice first show evidence of intraneuronal Aβ_1–42 _accumulation at ages when cognitive deficits begin to surface [19], and that as these mice age, they exhibit decreases in intraneuronal Aβ immunoreactivity with a concomitant increase in extracellular plaque load [26]. Historically, it has been technically difficult to detect intracellular Aβ with high confidence of specificity. To further detail the evolution of intracellular Aβ pathology in 3xTg-AD mice, we employed an immunohistochemical staining method optimized for visualization of intracellular Aβ_1–42 _peptide [27,28], which more readily unmasks intracellular Aβ peptide epitopes than standard formic acid epitope retrieval methods used for extracellular plaque immunohistochemistry. Using this methodology the employs a different anti-Aβ 42 antibody (Biosource/Invitrogen Aβ_1–42_), we have been able to reproducibly detect intracellular Aβ_1–42 _and demonstrated antibody specificity with cognate peptide competition tests (Figure 8A–D). The Covance/Signet 12F4 anti-Aβ_1–42 _antibody was not used with microwave pretreatment to detect intraneuronal Aβ. With formic acid pretreatment, the 12F4 antibody only labeled extracellular plaques and not Aβ localized intraneuronally. Evidence of intracellular Aβ_1–42 _immunopositivity using the Biosource/Invitrogen Aβ_1–42 _antibody was found in 3xTg-AD mouse brains beginning as early as 2 months of age (Figure 8E, M, U), and stably present throughout the time points assessed. The majority of intracellular Aβ_1–42 _expressing cells were detected outside of the pyramidal layer of the hippocampus, and found within the stratum oriens of the hippocampus (Figure 8E–T), the subiculum (Figure 8U-AB), and the corpus callosum (Figure 8M–T).

### Progression of Tau Pathology

Tau, which is expressed as 6 soluble isoforms from a genetic locus found on chromosome 17, is a microtubule-associated protein with numerous functions within the neuron [29-31]. One such cellular role is its ability to stabilize and promote the polymerization of microtubules [32-35]. This function has led to the hypothesis that the inability of tau to adequately bind and promote polymerization of microtubules would result in diminished transport within a neuron. Since it has been shown that the abnormal morphologic entity in AD brains known as the neurofibrillary tangle is comprised primarily of tau [36], it has been proposed that abnormalities of tau, directly or indirectly, play a central role in the pathogenesis of AD by progressively leading to a loss of fast axonal transport. A number of abnormalities of tau have been identified or suggested in AD neurons. These abnormalities include formation of tau into abnormal straight filaments or paired helical filaments [37-39], aggregations of paired helical filaments into the larger entities known as the neurofibrillary tangles (NFTs) [40], hyperphosphorylated tau [41-43], truncated tau [44], and the inability of tau to bind microtubules due to phosphorylation of key epitopes within the binding domain [45,46]. The human tau^P301L ^mutation, which is included as one of the transgenes harbored in 3xTg-AD mice, is commonly used in mouse models for studying human tauopathies, such as progressive supranuclear palsy, corticobasal degeneration, and frontal temporal dementia (reviewed in [47]). In these mouse models, intraneuronal inclusions of tau arise as a result of a number of progressive phosphorylation events on serine, threonine, and tyrosine residues [31,48], and eventually evolve into NFTs.

**Figure 9:**
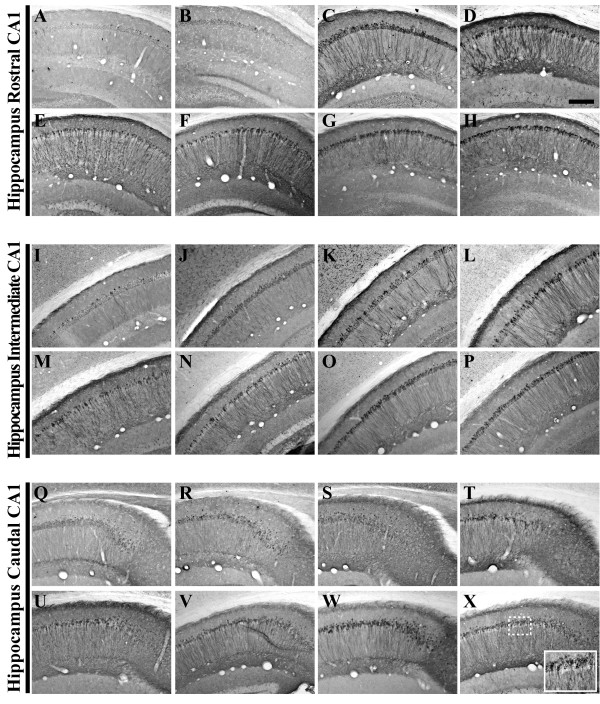
**Human tau^P301L ^transgene expression exhibits regionally and temporally disparate staining patterns in the 3xTg-AD mouse hippocampus**. Coronal mouse brain sections (30 μm) were prepared from 3xTg-AD mice sacrificed at 2 (**A, I, Q**), 3 (**B, J, R**), 6 (**C, K, S**), 9 (**D, L, T**), 12 (**E, M, U**), 15 (**F, N, V**), 18 (**G, O, W**), and 26 months of age (**H, P, X**) and were processed for immunohistochemistry using the HT7 monoclonal antibody to detect human tau^P301L ^transgene expression. CA1 hippocampal sections at Bregma -1.8 mm (**A–H**), at Bregma -2.5 mm (**I–P**), and at Bregma -2.8 mm (**Q–X**), were examined for regional and temporal patterns of human tau^P301L ^and photomicrographs were obtained at 10×. The inset in panel **X **represents a 40× digitally magnified image of the photomicrograph for better visualization of stained cell morphology. Scale bar in **D **represents 200 μm.

**Figure 10:**
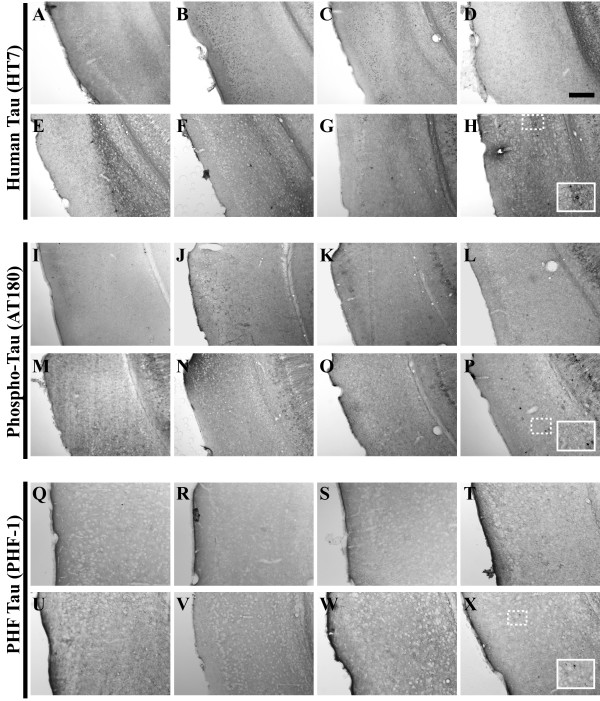
**Tau pathological hallmarks do not arise in the entorhinal cortex until 26 months of age**. Coronal mouse brain sections (30 μm) were prepared from 3xTg-AD mice sacrificed at 2 (**A, I, Q**), 3 (**B, J, R**), 6 (**C, K, S**), 9 (**D, L, T**), 12 (**E, M, U**), 15 (**F, N, V**), 18 (**G, O, W**), and 26 months of age (**H, P, X**) and were processed for immunohistochemistry to detect the human tau P301L mutant transgene product using the HT7 monoclonal antibody (**A–H**), human phospho-tau (Thr231) using the AT180 monoclonal antibody (**I–P**), and paired helical filament pathology using the PHF-1 monoclonal antibody (**Q–X**). Entorhinal cortex was examined for patterns of immunopositivity and photomicrographs were obtained at 10×. The insets in panels **H**, **P**, and **X **represent a 40× digitally magnified images of designated photomicrographs for better visualization of immunopositive cell/pathology. Scale bar in **D **represents 200 μm.

**Figure 11:**
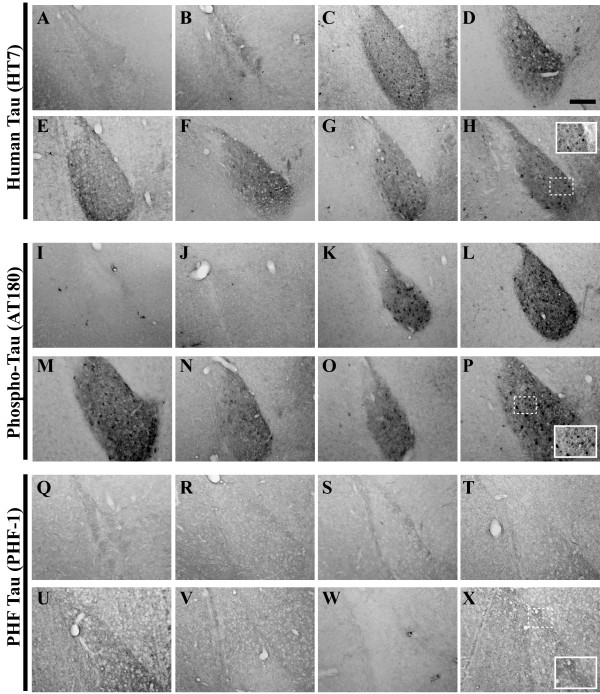
**The phospho-tau (Thr231) epitope is readily detectable, but paired helical filament pathology is virtually absent in the amygdala of 3xTg-AD mice**. Coronal mouse brain sections (30 μm) were prepared from 3xTg-AD mice sacrificed at 2 (**A, I, Q**), 3 (**B, J, R**), 6 (**C, K, S**), 9 (**D, L, T**), 12 (**E, M, U**), 15 (**F, N, V**), 18 (**G, O, W**), and 26 months of age (**H, P, X**) and were processed for immunohistochemistry to detect the human tau P301L mutant transgene product using the HT7 monoclonal antibody (**A–H**), human phospho-tau (Thr231) using the AT180 monoclonal antibody (**I–P**), and paired helical filament pathology using the PHF-1 monoclonal antibody (**Q–X**). Amygdala was examined for patterns of immunopositivity and photomicrographs were obtained at 10×. The insets in panels **H**, **P**, and **X **represent a 40× digitally magnified images of designated photomicrographs for better visualization of immunopositive cell/pathology. Scale bar in **D **represents 200 μm.

**Figure 12:**
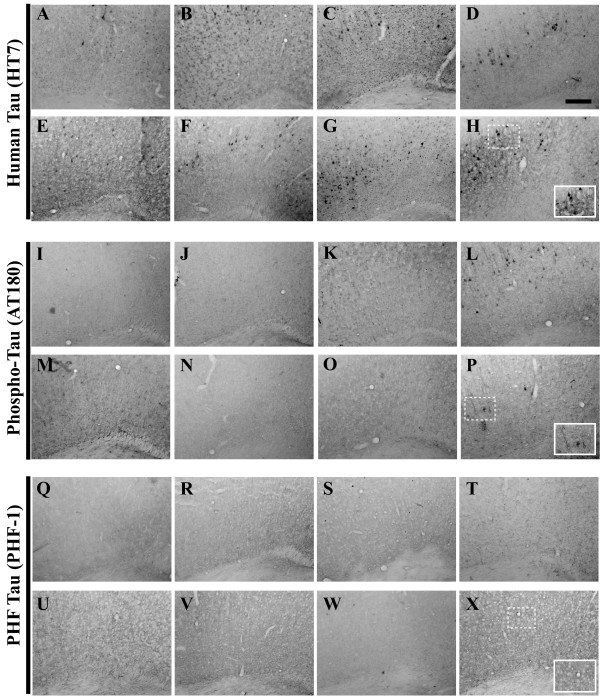
**Tau pathological hallmarks exhibit disparate staining patterns in the primary motor cortex of 2–26 month-old 3xTg-AD mice**. Coronal mouse brain sections (30 μm) were prepared from 3xTg-AD mice sacrificed at 2 (**A, I, Q**), 3 (**B, J, R**), 6 (**C, K, S**), 9 (**D, L, T**), 12 (**E, M, U**), 15 (**F, N, V**), 18 (**G, O, W**), and 26 months of age (**H, P, X**) and were processed for immunohistochemistry to detect the human tau P301L mutant transgene product using the HT7 monoclonal antibody (**A–H**), human phospho-tau (Thr231) using the AT180 monoclonal antibody (**I–P**), and paired helical filament pathology using the PHF-1 monoclonal antibody (**Q–X**). Primary motor cortex was examined for patterns of immunopositivity and photomicrographs were obtained at 10×. The insets in panels **H**, **P**, and **X **represent 40× digitally magnified images of designated photomicrographs for better visualization of immunopositive cell/pathology. Scale bar in **D **represents 200 μm.

Human tau^P301L ^transgene product can be detected using the HT7 antibody in a very limited number of 3xTg-AD pyramidal neurons in the CA1 of the hippocampus starting at 2 months of age (Figure 9A, I, Q). It was not until 6 months of age that a majority of pyramidal neurons harbored immunohistochemically detectable human tau. At both the 6 and 9-month time points (Figure 9C, K, S and 9D, L, T, respectively), the staining of axonal projections extending into the stratum radiatum began to intensify. Qualitatively, the staining for human tau^P301L ^transgene product in cell bodies and processes appeared to diminish starting at 12 months of age (Figure 9E, M, U), remaining relatively constant at subsequent ages. Such changes in apparent levels of human tau protein could correspond to age-related increases in the phosphorylation state of tau, leading to steric hindrance and/or structural alteration of the HT7 epitope. When we examined the entorhinal cortex, HT7-positive cells were not detectable until 12 months of age (Figure 10E), and no qualitative signs of human tau transgene product accumulation, as determined by intensification of immunopositive signal, were evident as the ages of the mice increased (Figure 10F, G, H). As in the case for hAPP^swe ^transgene expression, the human tau^P301L ^transgene product was detectable in the amydala beginning at 6 months of age and its levels did not appear to significantly fluctuate as the 3xTg-AD mice aged (Figure 11A–H). Primary motor cortex appeared to be the earliest of those examined to exhibit human tau^P301L ^transgene expression, where HT7-stained neurons were consistently detectable starting at 3 months of age (Figure 12A–H).

**Figure 13:**
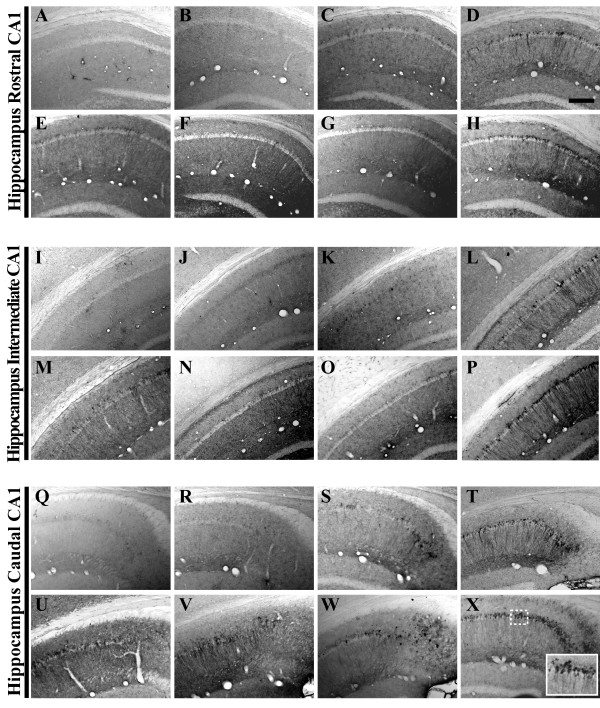
**The phospho-tau epitope Thr231 is immunohistochemically detectable in 3xTg-AD mouse hippocampus by 6 months of age**. Coronal mouse brain sections (30 μm) were prepared from 3xTg-AD mice sacrificed at 2 (**A, I, Q**), 3 (**B, J, R**), 6 (**C, K, S**), 9 (**D, L, T**), 12 (**E, M, U**), 15 (**F, N, V**), 18 (**G, O, W**), and 26 months of age (**H, P, X**) and were processed for immunohistochemistry using the AT180 monoclonal antibody to detect phospho-tau (Thr231) expression. CA1 hippocampal sections at Bregma -1.8 mm (**A–H**), at Bregma -2.5 mm (**I–P**), and at Bregma -2.8 mm (**Q–X**), were examined for regional and temporal patterns of human phospho-tau and photomicrographs were obtained at 10×. The inset in panel **X **represents a 40× digitally magnified image of the photomicrograph for better visualization of stained cell morphology. Scale bar in **D **represents 200 μm.

Oddo and colleagues recently showed evidence that numerous phospho-tau epitopes are immunohistochemically detectable in 3xTg-AD mice by 15 months of age [49]. To assess the status of one important phospho-tau epitope not examined in that prior report and its evolution in the 3xTg-AD mouse brain as a function of age, we examined the phosphorylation of tau at residue Thr231 using the AT180 antibody. We could detect a limited number of immunopositive neurons in the pyramidal layer of the hippocampus as early as 6 months of age (Figure 13C, K), with a majority of AT180-positive cells residing in more caudal regions (Figure 13S). Robust AT180 positivity was apparent at 9 months of age in cells of the pyramidal layer, and fibers extending into the stratum radiatum of the hippocampus (Figure 13D, L, T). As with the HT7 detection there appears to be a waning of AT180 staining at 12 months. However, at more advanced ages (26 months), AT180-positive signals re-intensified, suggesting that these mice could exhibit a cycling phenomenon of tau phosphorylation profiles. AT180-positive cells were not detectable until 26 months in the entorhinal cortex of 3xTg-AD mice (Figure 10P), strongly suggesting that these mice are not experimentally suitable for studying the effects of pathogenic tau in neuronal networks comprising this brain region. The amygdala stains with AT180 beginning at 6 months and this immunopositivity remains high at later ages (Figure 11K–P), whereas the primary motor cortex less consistently stained for this phospho-tau epitope (Figure 12I–P), in that immunopositive signal could be detected only at 9, 12, and 26 months of age.

**Figure 14:**
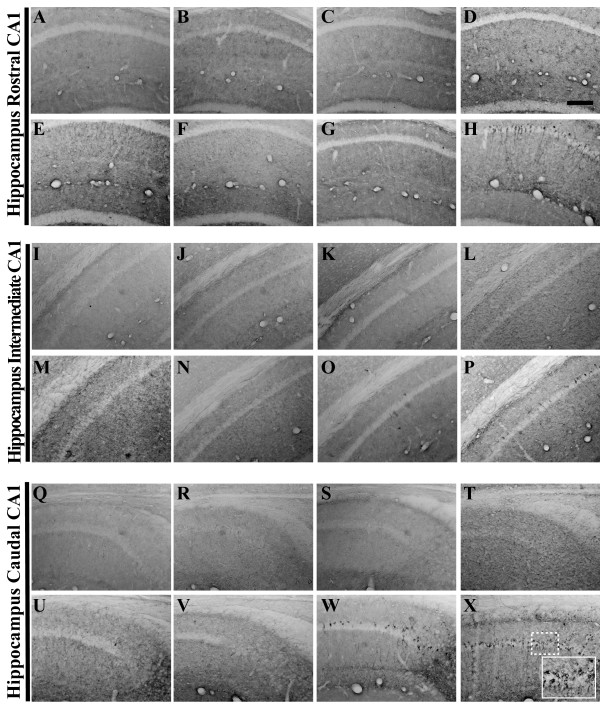
**Paired helical filament pathology does not arise until 18–26 months of age in 3xTg-AD mouse hippocampus**. Coronal mouse brain sections (30 μm) were prepared from 3xTg-AD mice sacrificed at 2 (**A, I, Q**), 3 (**B, J, R**), 6 (**C, K, S**), 9 (**D, L, T**), 12 (**E, M, U**), 15 (**F, N, V**), 18 (**G, O, W**), and 26 months of age (**H, P, X**) and were processed for immunohistochemistry using the PHF-1 monoclonal antibody to detect phospho-tau (Ser396 and Ser404) associated with paired helical filament pathology. CA1 hippocampal sections at Bregma -1.8 mm (**A–H**), at Bregma -2.5 mm (**I–P**), and at Bregma -2.8 mm (**Q–X**), were examined for regional and temporal patterns of PHF-1 immunopositivity and photomicrographs were obtained at 10×. The inset in panel **X **represents a 40× digitally magnified image of the photomicrograph for better visualization of stained cell morphology. Scale bar in **D **represents 200 μm.

As tau transitions to a more hyperphosphorylated state, it undergoes a self-assembly process into intertwining 4-nm paired helical filament (PHF) structures, further diminishing the ability of tau to preserve microtubule network integrity [50]. The monoclonal antibody PHF-1 (kindly provided by Dr. Peter Davies) recognizes PHF structural epitopes with robust affinity with trace reactivity towards unmodified normal human tau [51]. We were able to detect limited tau PHFs beginning in 15 month-old mice within the caudal CA1 region and subiculum (Figure 14W), but it was not until mice reached 26 months of age that we were able to consistently detect PHF-1 positive structures throughout the hippocampus (Figure 14H, P, X). Limited numbers of PHF-1 immunopositive cells were detectable in the entorhinal cortex and amygdala only at 26 months of age (Figures 10X and 11X), while PHF-1 immunopositive structures were barely detectable in primary motor cortex only at 26 months of age (Figure 12X).

### Age-related Patterns of Microglia and Astrocyte Staining

**Figure 15:**
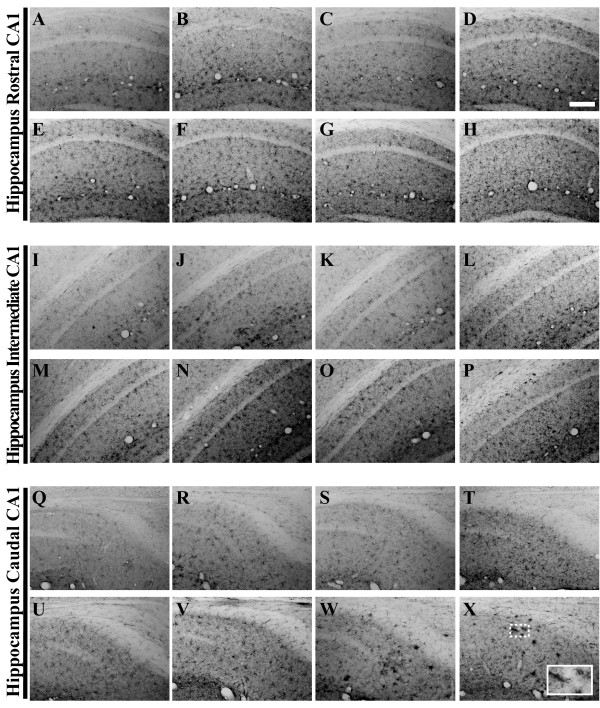
**Microglial staining patterns modulate as a function of age in the 3xTg-AD mouse hippocampus**. Coronal mouse brain sections (30 μm) were prepared from 3xTg-AD mice sacrificed at 2 (**A, I, Q**), 3 (**B, J, R**), 6 (**C, K, S**), 9 (**D, L, T**), 12 (**E, M, U**), 15 (**F, N, V**), 18 (**G, O, W**), and 26 months of age (**H, P, X**) and were processed for immunohistochemistry using the F4/80 monoclonal antibody to detect brain-resident microglia/macrophages. CA1 hippocampal sections at Bregma -1.8 mm (**A–H**), at Bregma -2.5 mm (**I–P**), and at Bregma -2.8 mm (**Q–X**), were examined for regional and temporal patterns of F4/80 immunopositivity and photomicrographs were obtained at 10×. The inset in panel **X **represents a 40× digitally magnified image of the photomicrograph for better visualization of stained cell morphology. Scale bar in **D **represents 200 μm.

**Figure 16:**
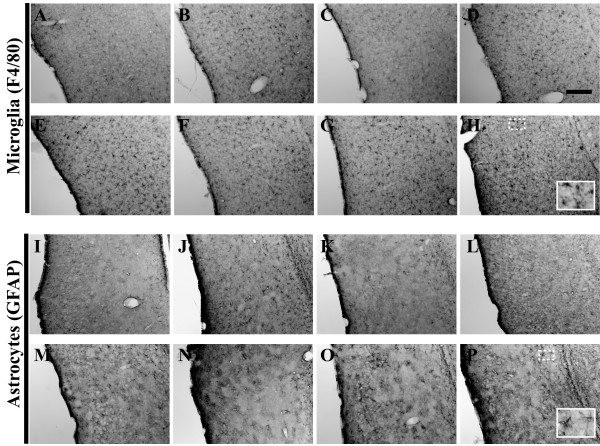
**Entorhinal cortex microglial and astrocytic staining patterns evolve on a similar timescale as observed in the 3xTg-AD hippocampus**. Coronal mouse brain sections (30 μm) were prepared from 3xTg-AD mice sacrificed at 2 (**A, I**), 3 (**B, J**), 6 (**C, K**), 9 (**D, L**), 12 (**E, M**), 15 (**F, N**), 18 (**G, O**), and 26 months of age (**H, P**) and were processed for immunohistochemistry to detect activated microglia using an anti-F4/80 specific monoclonal antibody (**A–H**) and astrocytes using an anti-GFAP specific monoclonal antibody (**I–P**). Entorhinal cortex was examined for patterns of immunopositivity and photomicrographs were obtained at 10×. The insets in panels **H **and **P **represent 40× digitally magnified images of designated photomicrographs for better visualization of immunopositive cells. Scale bar in **D **represents 200 μm.

**Figure 17:**
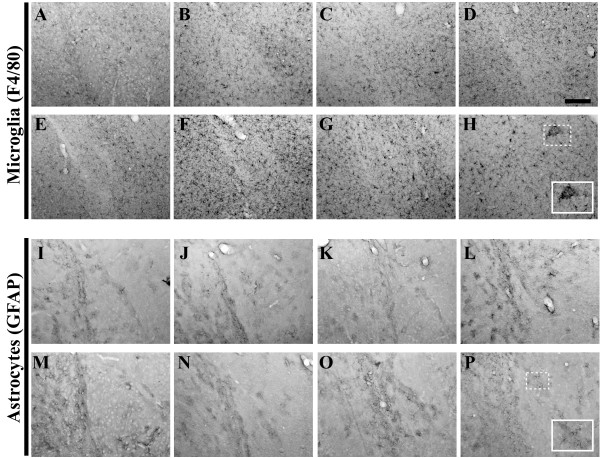
**Amygdala-resident microglial and astrocytic staining patterns evolve on a similar timescale as observed in the 3xTg-AD hippocampus**. Coronal mouse brain sections (30 μm) were prepared from 3xTg-AD mice sacrificed at 2 (**A, I**), 3 (**B, J**), 6 (**C, K**), 9 (**D, L**), 12 (**E, M**), 15 (**F, N**), 18 (**G, O**), and 26 months of age (**H, P**) and were processed for immunohistochemistry to detect activated microglia using an anti-F4/80 specific monoclonal antibody (**A–H**) and astrocytes using an anti-GFAP specific monoclonal antibody (**I–P**). Amygdala was examined for patterns of immunopositivity and photomicrographs were obtained at 10×. The insets in panels **H **and **P **represent 40× digitally magnified images of designated photomicrographs for better visualization of immunopositive cells. Scale bar in **D **represents 200 μm.

**Figure 18:**
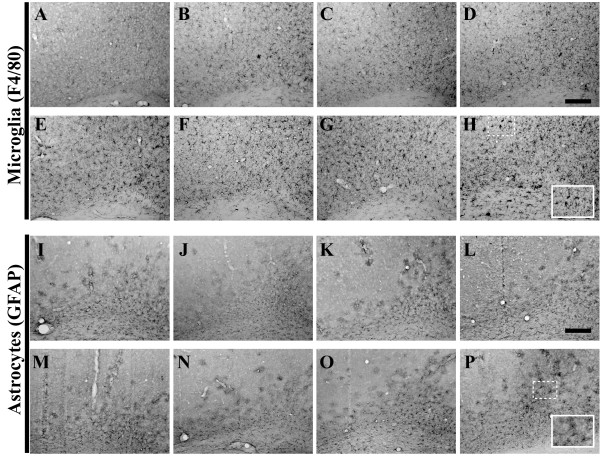
**Primary motor cortex microglial and astrocytic staining patterns evolve on a similar timescale as observed in the 3xTg-AD hippocampus**. Coronal mouse brain sections (30 μm) were prepared from 3xTg-AD mice sacrificed at 2 (**A, I**), 3 (**B, J**), 6 (**C, K**), 9 (**D, L**), 12 (**E, M**), 15 (**F, N**), 18 (**G, O**), and 26 months of age (**H, P**) and were processed for immunohistochemistry to detect activated microglia using an anti-F4/80 specific monoclonal antibody (**A–H**) and astrocytes using an anti-GFAP specific monoclonal antibody (**I–P**). Primary motor cortex was examined for patterns of immunopositivity and photomicrographs were obtained at 10×. The insets in panels **H **and **P **represent 40× digitally magnified images of designated photomicrographs for better visualization of immunopositive cells. Scale bar in **D **represents 200 μm.

Inflammatory processes have long been posited as serving integral roles in initiating and/or propagating AD-associated pathology within the human brain, as the elaboration of inflammatory cytokine expression and other markers of inflammation is more pronounced in individuals with known AD pathology. We previously reported significant enhancement of pro-inflammatory cytokine and chemokine expression and concomitant increases in region-specific microglial cell numbers, prior to the onset of overt amyloid pathology in young 3xTg-AD mice [52]. Herein, we assessed the status of two abundant non-neuronal cells traditionally activated in the setting of AD: microglia and astrocytes. The role of microglia and their accumulation at the sites of dense neuritic plaques has been described [53-55]. Immunohistochemical analysis of 3xTg-AD hippocampal brain tissue using an antibody specific for the microglia/macrophage surface marker, F4/80, revealed a qualitative enhancement of microglia staining from 2 (Figure 15A, I, Q) to 3 months of age (Figure 15B, J, R). The pattern of microglial distribution appeared rather uniform throughout the hippocampus from 3 to 15 months of age (Figure 15). Beginning at 18 months in the most caudal sections of hippocampus (Figure 15W) and continuing at 26 months (Figure 15X), there was a marked change in the distribution of microglia, with these cells appearing to assemble into dense aggregates, reminiscent of amyloid plaque-like structures. Microglial staining patterns in the entorhinal cortex were similar to those in hippocampus, except that aggregation of F4/80-positive cells was not overtly evident in 18 and 26 month-old 3xTg-AD mice (Figure 16A–H). F4/80-positive microglia in both the amygdala and primary motor cortex exhibited age-related distributions similar to those of the hippocampal formation (Figures 17A–H and 18A–H, respectively).

**Figure 19:**
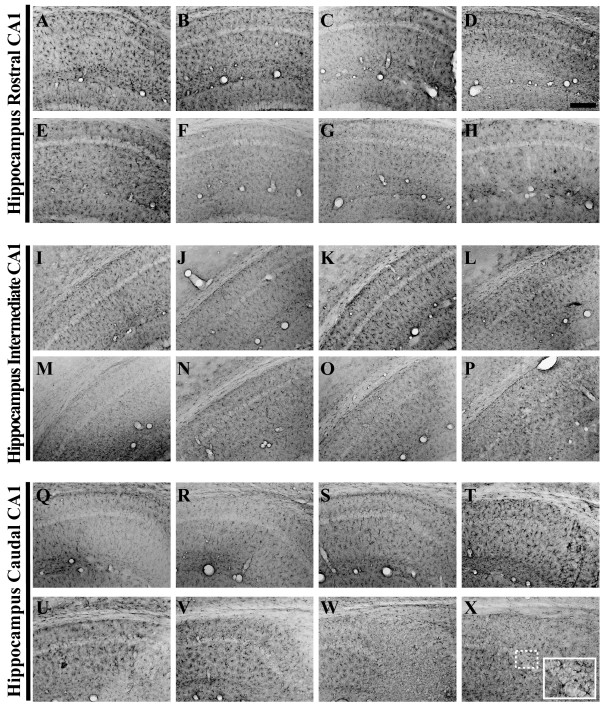
**GFAP-positive astrocyte staining remains relatively constant as a function of age in 3xTg-AD mouse hippocampus**. Coronal mouse brain sections (30 μm) were prepared from 3xTg-AD mice sacrificed at 2 (**A, I, Q**), 3 (**B, J, R**), 6 (**C, K, S**), 9 (**D, L, T**), 12 (**E, M, U**), 15 (**F, N, V**), 18 (**G, O, W**), and 26 months of age (**H, P, X**) and were processed for immunohistochemistry using a GFAP-specific monoclonal antibody to detect brain-resident astrocytes. CA1 hippocampal sections at Bregma -1.8 mm (**A–H**), at Bregma -2.5 mm (**I–P**), and at Bregma -2.8 mm (**Q–X**), were examined for regional and temporal patterns of GFAP immunopositivity and photomicrographs were obtained at 10×. The inset in panel **X **represents a 40× digitally magnified image of the photomicrograph for better visualization of stained cell morphology. Scale bar in **D **represents 200 μm.

Astrocytes are involved in many different functions in the brain, including structural integrity of the blood brain barrier, support of neuronal synapses by ion regulation and removal of glutamate [56]. Although it is believed that they are not directly responsive to primary insults, astrocytes react to inflammatory events in the brain, relying upon pro-inflammatory molecules elaborated from activated microglia [55]. Once signaled to do so, astrocytes can perpetuate inflammatory events in the brain via expression of iNOS and the enzyme argininosuccinate synthetase [57,58]. Glial fibrillary acidic protein (GFAP) is often employed as a marker of astrocytic activation. GFAP-expressing astrocytes were readily visible in 3xTg-AD mice at 2 months of age throughout the hippocampus (Figure 19A, I, Q), with limited signs of activation in the entorhinal cortex, amygdala, and primary motor cortex at this age (Figures 16I, 17I, and 18I). There appeared to be a qualitative decline in staining intensity in rostral hippocampal regions beginning at 15 months of age (Figure 19F) and continuing through 26 months of age (Figure 19H). However, the overall pattern of activated astrocyte staining within the hippocampus remained relatively constant as a function of age. More robust GFAP-positive astrocyte staining in the entorhinal cortex was more apparent at the 18- and 26-month time points (Figure 16O, P), while GFAP staining was less detectable, but constant at ages greater than 18 months in amygdala and primary motor cortex (Figures 17O, P and 18O, P, respectively).

## Discussion and conclusion

The 3xTg-AD mouse serves as an informative preclinical model employed ever increasingly in the examination of mechanisms underlying AD, as well as for the vetting of experimental AD-modifying therapeutics. The present study was designed to immunohistochemically document the evolution of transgene expression, amyloid deposition, pathogenic tau phosphorylation, astrogliosis, and microglial activation throughout the hippocampus and entorhinal cortex over a 26-month period in 3xTg-AD mice. A summary of pathological progression and qualitative severity scoring in these mice is illustrated in Table 2. Previously published reports employing the 3xTg-AD model have primarily focused upon mechanisms/pathologies pertaining to either early or late-stage disease, and have provided limited insight into the overall progression of evolving AD-related pathologies using a systematic immunohistochemical approach. Gaining a detailed understanding as to how hAPP^swe ^and tau^P301L ^transgene expression relates temporally and spatially to the appearance of pathogenic Aβ peptide- and hyperphosphorylated tau-related pathologies enables the informed design and implementation of future studies. Moreover, given that these AD-related pathologies exhibit subtle regional differences within the 3xTg-AD mouse brain, the information gleaned from systematic immunohistochemical assessment hones the focus of endpoint analyses on brain regions more or less severely impacted depending upon the hypotheses being tested.

**Table 2:** Qualitative assessment of pathological progression by region in male 2–26 month-old 3xTg-AD mice.

**Antigen**	**Age (months)**							
**hAPP**	**2**	**3**	**6**	**9**	**12**	**15**	**18**	**26**
Primary motor cortex	+	+	+	+	+	+	+	+
Amygdala	-	-	+	+	++	++	++	++
Rostral Hippocampus CA1	+	+	+	+	++	++	++	++
Medial Hippocampus CA1	+	+	+	+	++	++	++	++
Caudal Hippocampus CA1/Subiculum	+	+	+	+	++	++	++	++
Entorhinal Cortex	+	+	+	+	++	++	++	++

**hAPP/Aβ**								
Primary motor cortex	+	+	++	++	++	++	+++	+++
Amygdala	+	+	++	++	++	++	+++	+++
Rostral Hippocampus CA1	-	-	+	+	++	++	+++	+++
Medial Hippocampus CA1	-	-	+	+	++	++	+++	+++
Caudal Hippocampus CA1/Subiculum	-	+	+	+	++	+++	+++	+++
Entorhinal Cortex	-	-	+	+	+	+	+++	+++

**hAβ 1–42 (Extracellular)**								
Primary motor cortex	-	-	-	-	-	-	+	++
Amygdala	-	-	-	-	-	-	++	+++
Rostral Hippocampus CA1	-	-	-	-	-	-	+	++
Medial Hippocampus CA1	-	-	-	-	-	-	+	++
Caudal Hippocampus CA1/Subiculum	-	-	-	-	-	+	++	+++
Entorhinal Cortex	-	-	-	-	-	-	+	+++

**hAβ 1–42 (Intracellular)**								
Primary motor cortex	+/-	+/-	+	+	++	++	++	++
Amygdala	+/-	+/-	+	+	++	++	++	++
Rostral Hippocampus CA1	+/-	+	++	++	++	++	++	++
Medial Hippocampus CA1	+	++	++	++	++	++	++	++
Caudal Hippocampus CA1/Subiculum	+	++	++	++	++	++	++	++
Entorhinal Cortex	+/-	+	+	+	+	+	+	+

**Human Tau**								
Primary motor cortex	+/-	+	+	+	+	+	+	+
Amygdala	-	-	+	+	+	+	+	+
Rostral Hippocampus CA1	+/-	+/-	+	+	+	+	+	+
Medial Hippocampus CA1	+/-	+/-	+	+	+	+	++	++
Caudal Hippocampus CA1/Subiculum	+/-	+/-	+	+	+	+	++	++
Entorhinal Cortex	-	-	-	-	-	+/-	+/-	+/-

**Phospho-hTau (Thr231)**								
Primary motor cortex	-	-	-	+	+	+/-	+/-	+
Amygdala	-	-	+	++	++	+	+	++
Rostral Hippocampus CA1	-	-	+/-	+	+	+/-	+/-	+
Medial Hippocampus CA1	-	-	+/-	+	+	+	+/-	++
Caudal Hippocampus CA1/Subiculum	-	-	+	+	+	+	+	+
Entorhinal Cortex	-	-	-	-	-	-	-	+/-

**Paired Helical Filaments**								
Primary motor cortex	-	-	-	-	-	-	-	-
Amygdala	-	-	-	-	-	-	-	-
Rostral Hippocampus CA1	-	-	-	-	-	-	+/-	+
Medial Hippocampus CA1	-	-	-	-	-	-	+/-	+
Caudal Hippocampus CA1/Subiculum	-	-	-	-	-	-	+	+
Entorhinal Cortex	-	-	-	-	-	-	-	+/-

**Microglia**								
Primary motor cortex	+	+	+	+	+	+	++	++
Amygdala	+	+	+	+	+	+	+	++
Rostral Hippocampus CA1	+	+	++	++	++	++	++	++
Medial Hippocampus CA1	+	+	++	++	++	++	++	++
Caudal Hippocampus CA1/Subiculum	+	+	++	++	++	++	+++	+++
Entorhinal Cortex	+	+	++	++	++	++	++	++

**Astrocytes**								
Primary motor cortex	+	+	+	+	+	+	+	+
Amygdala	+/-	+/-	+/-	+/-	+/-	+/-	+/-	+/-
Rostral Hippocampus CA1	+	+	+	+	+	+	+	+
Medial Hippocampus CA1	+	+	+	+	+	+	+	+
Caudal Hippocampus CA1/Subiculum	+	+	+	+	+	+	+	+
Entorhinal Cortex	+	+	+	+	+	+	+	+

We found that patterns of intracellular Aβ peptide immunoreactivity do not correlate with the patterns of human APP^swe ^transgene expression in 3xTg-AD mice. Both Aβ_1–42_-specific antibodies that were employed, which have been pre-absorbed to eliminate binding to non-Aβ_1–42 _species, exhibit distinct patterns of staining that lie outside of the pyramidal cell layer of the hippocampus and layer II/III of the entorhinal cortex. These cell layers, however, show heavy immunoreactivity for human APP^swe ^transgene product. A number of groups have demonstrated that APP is synthesized at the cell body and undergoes anterograde axonal transport to distal compartments where it is ultimately proteolytically processed (reviewed in [59]). Neurons emanating from layer III of the entorhinal cortex extend bilaterally into the CA1 and CA3 of the hippocampus and subiculum [60], whereas pyramidal neurons from the CA1 of the hippocampus project into layer V of the entorhinal cortex and to areas of the subiculum [61]. This evidence taken together with the observed patterns of hAPP^swe ^transgene expression within cells that comprise the perforant pathway in 3xTg-AD mice, it is reasonable to propose that the Aβ_1–42 _cleavage product is generated and accumulates at termini.

We should note that the cells harboring intracellular Aβ are purported to be neurons, but whether they represent the sole cell type physiologically influenced by intracellular Aβ peptide accumulation is unlikely. It is known that other cell types [62], including microglia [53] and astrocytes [63], are able to pinocytize and phagocytize extracellular Aβ_1–42_. These cells may represent viable targets for the deleterious effects attributed to intracellular Aβ peptides. Cummings and colleagues demonstrated previously that Aβ peptides, produced initially by neurons and deposited at neuronal terminals, are readily able to form higher order aggregates [64]. This may also explain the spatial alterations found with in the hippocampus where plaque burden is observed at earlier ages in the more caudal regions of the 3xTg-AD brain. Hence, the anatomical interconnectivity of these projections and biogenesis and subsequent proteolytic processing of hAPP^swe ^likely underlie the dissonant patterns of Aβ and hAPP^swe ^immunoreactivity observed in the CA1, subiculum, and the entorhinal cortex of 3xTg-AD mice.

It was somewhat surprising that extracellular Aβ_1–42 _plaques were not readily detectable in male 3xTg-AD mice in our study until 15 months in the caudal hippocampus and 18 months in cortical structures, findings that are in conflict with Oddo and colleagues that stated in their original 2003 report that 3xTg-AD mice exhibit amyloid plaques beginning at 6 months of age in the frontal cortex [17]. These disparate results may be the result of the antibodies employed or the genders of mice studied in each study, but such details are absent from the original report making it difficult to resolve this issue. Other explanations may exist, including loss of phenotype due to a progressive loss of transgene copies with successive breeding, different founder/line of mice provided to outside investigators compared to the mice described in the original report, as well as differences in housing conditions. The mice used in the present study were pathogen-free and were maintained in a pathogen-free facility throughout the duration of the experiments. Moreover, we have not observed any overt reduction in transgene copy numbers over the 12+ generations we have housed 3xTg-AD mice within our animal colony. However, determining the differences in pathological progression and elucidating the underlying cause(s) is important for generation of consistent data sets across different laboratories that are meaningful and provide generally applicable mechanistic insight into AD-related processes.

Similar to the hAPP^swe ^transgene product, human tau^P301L^, as detected with the HT7 antibody, appears immunohistochemically at 2–3 months of age in 3xTg-AD mice. This is not surprising given that both hAPP^swe ^and tau^P301L ^transgenes are transcriptionally controlled by the Thy 1.2 promoter, and that the transgenes are genetically linked due to the method by which the 3xTg-AD mice were derived [17]. Using phospho-epitope specific antibodies, Sahara and colleagues showed that tau proceeds through a series of post-translational phosphorylation events that progressively lead to increased insolubility and decreased functionality [31]. We were able to detect phosphorylation at the Thr231 amino acid residue of tau, an indication of pathogenic progression, as early as 6 months of age in cells residing within the pyramidal layer of the hippocampus. This evidence suggests that tau dysfunction contributes to AD-related pathophysiology in 3xTg-AD mice at ages earlier than purported in previously published studies [18]. Oddo and colleagues reported that the Thr231 phospho-epitope of tau is not detectable until 12 months of age. Given our findings, designs of future studies seeking to dissect the differential influences of APP^swe ^and tau^P301L ^on brain physiology need to take into account the overlapping temporal expression patterns of the 3xTg-AD transgenes.

We have previously demonstrated that various inflammatory events correspond to the presentation of early (< 6 months of age) intracellular Aβ pathology in 3xTgAD mice [52]. Specifically, the pro-inflammatory cytokine tumor necrosis factor-alpha (TNF-α) and chemokine monocyte chemoattractant protein-1 (MCP-1) are expressed at heightened levels in a region-specific pattern, and these enhanced molecule expression profiles correlate with increased numbers of microglia specifically within 3xTg-AD mice as compared to age-matched, non-transgenic control mice. As such, it was imperative in the present study to continue to monitor alterations in glial marker expression as these mice age as staining intensity and/or pattern changes could provide insight into pathophysiology. We observed a clustering of F4/80-positive microglia/monocyte cells within areas exhibiting heavy amyloid burden in 3xTg-AD mice at 15 to 26 months of age. It is likely that this pattern of microglial accumulation is indicative of association with Aβ-containing plaques similar to that observed in human AD brain. Interestingly, 3xTg-AD mice do not show noticeable age-related enhancement GFAP-positive astrocyte staining intensities, which markedly differentiates this model from what has been documented in other AD mouse models, including the PDAPP mouse [65]. These differences in activation of astrocytes may inherently relate to the relative strength and/or cell type expression specificities of the promoters employed to drive each of the respective AD-related transgenes (reviewed by [66]).

Other investigators have shown that amyloidogenic mouse models of AD exhibit gender-related differences in severity of pathology [67]. In this study we present a time course of AD-related pathological progression solely for male 3xTg-AD mice in order to more finely assess intermediate ages for subtle region-specific differences. While Clinton et al. have shown that sexual dimorphisms in cognition and stress responses are apparent between male and female 3xTgAD mice [68], that study did not include an extensive histological comparison of brains from the two genders. Carroll and colleagues more recently reported that female 3xTg-AD mice exhibit an earlier onset of AD pathology and this may be a consequence of progesterone and estrogen-mediated signaling mechanisms [69]. Given these gender-specific differences in time of onset and severity of behavioral phenotype it is imperative that future experimental therapeutics be vetted in both male and female 3xTg-AD mice. Moreover, the underlying neuroinflammatory state of each gender is likely disparate and may markedly impact the efficacy and/or safety profile of a particular therapeutic, especially if that modality is immune-based in nature. The 3xTg-AD model provides an informative platform on which to test new therapeutic modalities, but the regional, temporal, and gender-specific subtleties and limitations of this model must be fully appreciated before this model is elevated to "golden standard" status in the field of AD research.

## Methods

### Transgenic Mice

The 3xTg-AD mice (B1 line) were kindly provided by Frank LaFerla (University of California, Irvine; [17]). All mice were housed and bred in accordance with University of Rochester requirements for animal welfare and care. Homozygous 3xTg-AD mice were monogamously mated to produce offspring, which were housed until sacrificed at the designated age. Mice were sacrificed via pentabarbitol overdose and subsequently transcardiac perfused with heperanized saline, followed by 4% paraformaldehyde in 0.1 M phosphate buffer (PB). Brains were removed and post-fixed overnight in 4% paraformaldehyde in 0.1 M PB, followed by equilibration in 20% sucrose in 0.1 M phosphate-buffered saline (PBS) and then 30% sucrose in 0.1 M PBS. Brains were coronally sectioned on a freezing stage sliding microtome (Microm, Walldorf, Germany) at 30 μm and stored in cryoprotectant at -20°C until immunohistochemical processing.

### Nissl Staining

Brain sections were washed with 0.15 M PB for 2 h to remove the cryoprotectant, and mounted on Superfrost^® ^Plus slides (VWR International, West Chester, PA) and allowed to dry completely. The slides were subsequently hydrated in dH_2_0 for 5 min. before being stained with 0.02% Cresyl violet Acetate in 0.25% Acetic acid for 30 min. Sections were rinsed in 3 changes of dH_2_0, and placed in 50% ethanol for 1 min. followed by 70% for 1 min. to destain. Sections were allowed to dry and then cleared by dipping in xylene before being coverslipped.

### Antibodies

The following antibodies were used at the designated working dilutions: anti-amyloid precursor protein A4, corresponding to the NPXY motif of hAPP, (Clone Y188; AbCam, Cambridge, MA, 1:750); anti-hAPP/amyloid-beta reactive to amino acid residue 1–16 of beta-amyloid (6E10; Covance, Berkeley, CA; 1:1000); anti-amyloid beta 1–42 clone 12F4 reactive to the C-terminus of beta-amyloid and specific for the isoform ending at amino acid 42 (Covance/Signet, Berkeley, CA, 1:1000); anti-amyloid beta 1–42 polyclonal antibody for intracellular amyloid-beta staining (Invitrogen, Carlsbad, CA, formerly Biosource, Hopkinton, MA 1:1000); anti-human tau HT7, reactive to residues 159 to 163 (Pierce, Rockford, IL; 1:200); anti-human phosphorylated tau AT180, specific for htau phosphorylated at the Thr231 residue (Pierce, Rockford, IL; 1:200); anti-human phosphorylated tau PHF-1 (gift from Dr. Peter Davies, Albert Einstein College of Medicine; 1:30); anti-glial fibrillary acidic protein GFAP (Dako Cytomation, Glostrup, Denmark; 1:1000); and an antibody specific for the microglial/monocytic cell surface marker F4/80 (AbD Serotec, Raleigh, NC; 1:500). Specificity of the anti-Aβ_1–42 _antibodies (12F4 from Covance/Signet for extracellular Aβ staining and anti-Aβ_1–42 _polyclonal from Biosource/Invitrogen for intracellular Aβ staining) was confirmed using peptide competition experiments. The results of these assessments are illustrated in Figures 7 and 8, respectively.

### Immunohistochemistry

Brain sections were washed with 0.15 M PB for 2 h to remove the cryoprotectant, then incubated with 3% H_2_O_2 _in 0.15 M PB for 20 min. to quench endogenous peroxidase activity. For Aβ peptide-specific stains, the sections were treated with 70% formic acid for 15 min. for epitope retrieval. For the intracellular Aβ_1–42_stain, we employed a microwave/Target buffer (Dako Cytomation, Glostrup, Denmark) epitope retrieval method as described previously [27]. Briefly, the brain sections were washed and peroxidase activity quenched. The sections were mounted on to slides and allowed to dry. The Target buffer was heated to 98°C in a microwave (GE, Louisville, KY), the slides submerged into the buffer and placed in the microwave, twice for 3 min. at 450 W, and allowed to rest for 5 min. between microwave steps. Brain sections were similarly processed for immunohistochemistry as detailed below. The sections were washed and permeabilized in 0.15 M PB and 0.4% Triton X-100, followed by blocking in 0.15 M PB with 10% normal goat serum, and 0.4% Triton X-100. After blocking, the sections were incubated in 0.15 M PB with 1% normal goat serum, and 0.4% Triton X-100, with the designated primary antibody. The sections were washed with 0.15 M PB, followed by an incubation with the appropriate secondary biotin-conjugated secondary antibodies (Vector Labs, Burlingame, CA; 1:1000) in 0.15 M PB with 1% normal goat serum, and 0.4% Triton X-100. The sections were washed with 0.15 M PB with 1% normal goat serum and 0.4%Triton X-100, and incubated in the avidin-biotin complex (Vector Labs Vectastain ABC System as per manufacturer's protocol, Vector Labs, Burlingame, CA). Sections were washed in 0.15 M PB followed by rinses in dH_2_O. The sections were developed with nickel-enhanced DAB (Vector Labs, Burlingame, CA). Sections were mounted on Superfrost^®^Plus slides (VWR International, West Chester, PA) cover-slipped and viewed using an Olympus AX-70 microscope and motorized stage (Olympus, Center Valley, PA) and the MCID 6.0 Imaging software (Interfocus Imaging subsidiary of GE Healthcare, Cambridge, England).

### Qualitative scoring of immunohistochemical staining intensities

Time points were compared to one another within a particular antibody staining group. Images were analyzed using the relative optical density (ROD) score in the MCID 6.0 Imaging software (Interfocus Imaging subsidiary of GE Healthcare, Cambridge, England). Sections of all the mice, corresponding to the areas of study, were scored according to the following schema: (-) indicates no staining present, (+/-) indicates limited number of cells/structures showing evidence of staining, (+) denotes consistent expression of the marker, (++) represents an elevated expression measured in ROD of Δ 0.075 to 0.250, and (+++) represents a further increase in staining as indicated by a change in ROD of Δ 0.251 greater than (+) staining intensities.

## Abbreviations

AD: Alzheimer's disease; APP: amyloid precursor protein; APP^swe^: amyloid precursor protein Swedish mutation; PHF: paired helical filament; PS1: Presenilin 1; Aβ: Beta-amyloid; TNF-α: Tumor necrosis factor-alpha; MCP-1: monocyte chemoattractant protein-1; Tg: Transgenic; PB: Phosphate buffer; PBS: Phosphate-buffered saline.

## Competing interests

The authors declare that they have no competing interests.

## Authors' contributions

MAM performed brain sectioning and immunohistochemistry, and aided in the preparation of the manuscript. WJB conceived the design of the study, aided in the preparation of the manuscript, and provided critical analysis of the manuscript. Both authors read and approved the final manuscript.
